# How Does Ethylenediaminetetraacetic Acid Irrigation Affect Biodentine? A Multimethod Ex Vivo Study

**DOI:** 10.3390/ma17061230

**Published:** 2024-03-07

**Authors:** Katarzyna Dąbrowska, Aleksandra Palatyńska-Ulatowska, Leszek Klimek

**Affiliations:** 1 Department of Endodontics, Chair of Conservative Dentistry and Endodontics, Medical University of Lodz, 251 Pomorska Street, 92-217 Lodz, Poland; katarzyna.anna.dabrowska@umed.lodz.pl; 2 Institute of Materials Science and Technology, Technical University of Lodz, 1/15 Stefanowskiego Street, 90-924 Lodz, Poland; leszek.klimek@p.lodz.pl

**Keywords:** Biodentine, EDTA, energy dispersive spectroscopy, irrigation, perforation, scanning electron microscope, ultrasonic activation

## Abstract

The activity of biomaterials used during endodontic treatment can be affected by various factors. One of them is the chemical action of the irrigant that they are exposed to. The aim of this multimethod ex vivo study was to evaluate the influence of ethylenediaminetetraacetic acid (EDTA) on the surface appearance and chemical composition of Biodentine used in perforation repair. Twenty material specimens were prepared according to manufacturers’ recommendations and divided into two setting-time-based groups, tested after 45 min (group A) and 24 h (group B) of setting. Material was irrigated with 17% EDTA solution with or without simultaneous ultrasonic activation. The surface characteristics and the chemical composition analysis of the Biodentine specimens were performed with the aid of a scanning electron microscope (SEM) and an energy dispersive spectroscopy (EDS) method, respectively. The volumetric loss of material was measured by dedicated digital software in an optical microscope. Statistical analysis was performed. The EDS study confirmed that after the rinsing protocol, the percentage content of elements differed between the groups. The EDTA rinse, whether ultrasonically activated or not, visibly affected the surface appearance and chemical composition of Biodentine. The specimens’ surface subjected to irrigation was more irregular under SEM than in a control group. The US activation of the liquid amplified its impact on the tested material. The average volume loss in group A after 5 min irrigation was 3.98 µm^3^ for each µm^2^ of the chosen area and it increased up to 7.74 µm^3^/μm^2^ after the ultrasonic activation. In group B, indicated volume loss values were 6.30 and 11.70 µm^3^/μm^2^ for 5 min irrigation without and with US activation, respectively. Using a 20 min irrigation time and ultrasonic activation increased it up to 32.71 µm^3^/µm^2^. Each rinsing protocol involving irrigation with ethylenediaminetetraacetic acid modified the surface features and the chemical composition of the evaluated hydraulic tricalcium silicate cement. Further research is needed to indicate the possible impact of the observed changes on its long-term clinical performance.

## 1. Introduction

Complicated endodontic cases, such as chamber floor or furcal perforations, often require the use of highly specified materials, that should efficiently seal the defect and heal the wounded area without harming the contacting intraosseous environment [1]. For the last 25 years a Portland-cement-based mineral trioxide aggregate (MTA) has been used with success for this purpose [2]. The evolution of this material has led to the creation of the whole bioceramic group of dressing cements. Their therapeutic properties, biocompatibility and antimicrobial activity, all deriving from a specific chemical composition, are essential to ensure treatment success [3]. Nevertheless, proper material management is of equal importance. Hence, the clinicians’ ability to correctly choose and use these biocements needs to be evaluated and updated.

Biodentine (Septodont, Saint-Maur-des-Fosées, France), also known as “dentine replacement” material [4,5], is a representative of these reparative cements. It is a hydraulic self-setting tricalcium silicate cement with many clinical applications. Biodentine is used in direct and indirect pulp capping [6,7,8], pulpotomy procedures [9,10], perforation repair [2,11], treatment of immature teeth or an open apex [2,10], root-end retrograde surgical filling [2,12] and as a dentin replacement material [13]. Compared to MTA, it has a shorter setting time [2,14] and comparable healing and sealing properties [15]. Biodentine’s main components are di- and tricalcium silicates, which ensure its bioactivity [16]. The manufacturer also uses zirconium oxide as a radiopacifier, calcium chloride as a setting reaction accelerator, calcium carbonate and oxide as fillers, and a hydrosoluble polymer that acts as a water requirement reductor [17].

Standard endodontic procedure, together with mechanical shaping, involves a thorough chemical preparation with the use of 5.25% sodium hypochlorite (NaOCl), 2% chlorhexidine (CHX) and chelating agents—17% ethylenediaminetetraacetic acid (EDTA) or 40% citric acid (CA). This is a crucial part of the canal space disinfection. For the same reason, in more complicated cases of root canal or chamber perforations, constant irrigation following the placement of bioceramic material is required. Endodontic space perforation is a pathogenic or iatrogenic communication between the root canal system and the external tooth surface [18]. The prognosis largely depends on immediate and proper treatment [19]. It often requires the placement of the bioactive material in the first place, e.g., to stop bleeding. The following root canal treatment is performed after closure of the defect. Therefore, the material used for this procedure will be exposed to, among other things, chemical action of the irrigants used after its placement. The cement resistance depends on its setting stage in the moment of irrigation as well as the characteristics of the irrigant. Immediate irrigation may negatively affect the clinical performance of the biocement used for perforation closure [20,21].

This research includes ethylenediaminetetraacetic acid as a rinsing solution used in the final irrigation protocol. It is a chelator able to dissolve inorganic structures by binding calcium and magnesium ions and replacing them with sodium ions [22]. Although its chelating nature is highly required in removing the smear layer and exposing dentinal tubules for better sealer adaptation [23], it may pose a threat to any biocement exposed to it.

There are various irrigation techniques and methods to enhance the standard irrigation procedure. The most common is ultrasonic activation of the rinsing liquid, known as passive ultrasonic irrigation (PUI). The acoustic streaming produced at the tip of the device can remove debris from the canal walls and rupture any aggregations of bacteria [24]. This process is additionally supported by microcavitation and temperature rise in the irrigating solution [25].

The aim of this research was to analyze the influence of ethylenediaminetetraacetic acid used as an irrigant in endodontic procedures, either ultrasonically activated or not, on Biodentine surface appearance and its chemical composition. The volumetric loss of material was evaluated, which is a novelty of this study. Scarce literature on this topic makes the study the first of its kind. The null hypothesis of no differences between different irrigation protocols was adopted.

## 2. Materials and Methods

**Figure 1:**
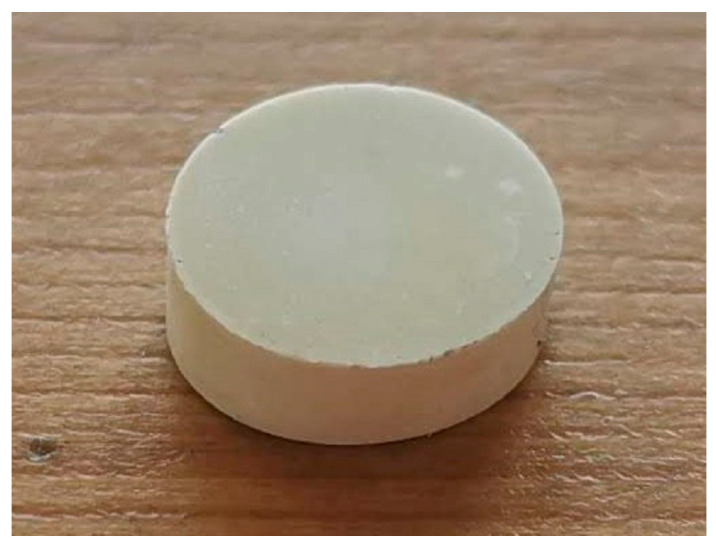
Cylindrical Biodentine specimen.

Biodentine cement was prepared following the manufacturer’s instructions. The powder–liquid ratio, mixing time and method were strictly respected: 5 drops of the dedicated liquid were added vertically to the capsule containing the powder. The capsule was closed and mixed for 30 s in a mixing device (Dental Mixer SYG200, Septodont, Saint-Maur-des-Fosées, France) at a speed of 4000 rpm. Mixed material was placed in polyvinyl tubes (polyvinyl chloride—PVC; Cellfast, Stalowa Wola, Poland) and compacted with the aid of a plugger on a smooth glass plate. The specimens were divided into two setting-time-based groups: group A—investigated after 45 min, group B—after 24 h of setting. After designated times, the material was cautiously removed from the forms. Biodentine specimens were then dry-polished with rotating sandpapers with subsequent grits from 600 to 1200 in order to equalize the investigated surfaces. Twenty standardized cylindrical specimens of 3 mm height and 8 mm diameter (Figure 1) were prepared this way.

**Figure 2:**
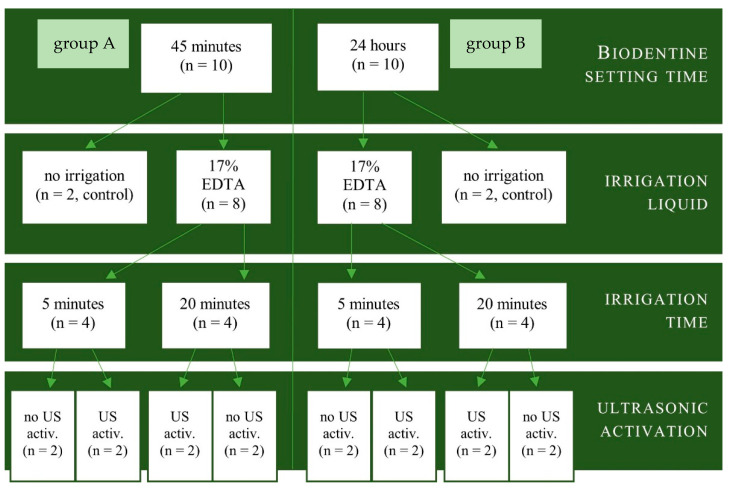
Biodentine specimens’ distribution.

The specimens were categorized randomly into groups, as shown in Figure 2.

The control group consisted of 4 specimens and was not subjected to any irrigation protocol. The research group was rinsed with 10 mL of 17% EDTA (Cerkamed, Stalowa Wola, Poland) for 5 or 20 min, with or without continuous ultrasonic activation in a Sonic-0.5 device (Polsonic, Warsaw, Poland). After each protocol, Biodentine specimens were immersed in demineralized water and lightly dried with compressed air in order to avoid EDTA remnants or any other precipitates on the investigated surfaces.

The surface appearance of each Biodentine specimen, after having been subjected to its designated irrigation protocol, was analyzed in the scanning electron microscope (SEM, S–3000N, HITACHI, Tokyo, Japan) with a magnification of 1.0 k, 15 kV accelerating voltage and 15 mm working distance on an area of 2 × 3 mm. The chemical composition of the material’s surface was examined in SEM using X-ray microanalysis with the energy dispersive spectroscopy method (EDS) with the aid of the Vantage software (Version 4.1, Thermo Fisher Scientific, Waltham, MA, USA) and AZtec software (Version 1.0A, Oxford Instruments, Tubney Woods, Abingdon, UK). The electron beam was automatically set so that the detector could keep up with counting the X-ray quanta. The detector dead time was set at 25–30%. Biodentine volumetric loss was evaluated in an optic microscope VHX-950F with its dedicated digital software (Keyence, Mechelen, Belgium) using 0.5 k magnification. The measurements were taken in 5 randomly chosen areas of each specimen and their arithmetic mean and standard deviation were calculated. Statistical analysis of the obtained results was carried out in order to compare the dependence of the performed rinsing protocol on the loss of material volume. As the specimens did not meet the normality assumption, the Kruskal–Wallis test was used in Statistica 13 software (StatSoft, Tulsa, OK, USA). A *p* value below 0.05 was deemed significant. The null hypothesis of no differences between different irrigation protocols was adopted.

## 3. Results

### 3.1. Biodentine Surface Chemical Composition

**Figure 3:**
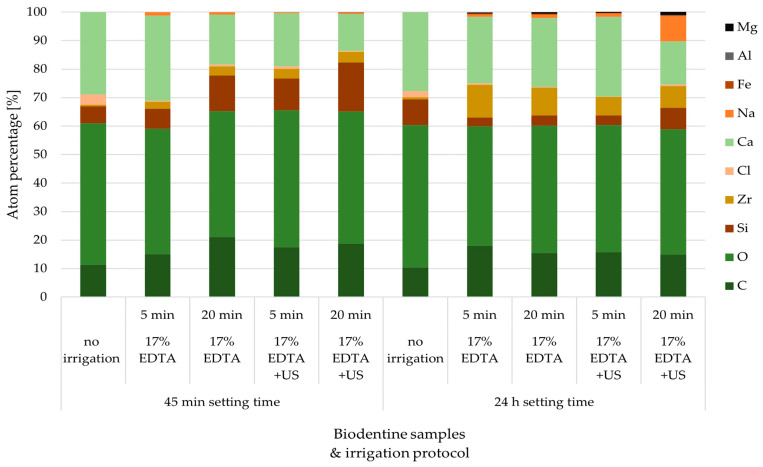
Changes in the chemical composition of Biodentine specimens’ surface after different irrigation protocols involving 17% sodium edetate solution.

**Figure 4:**
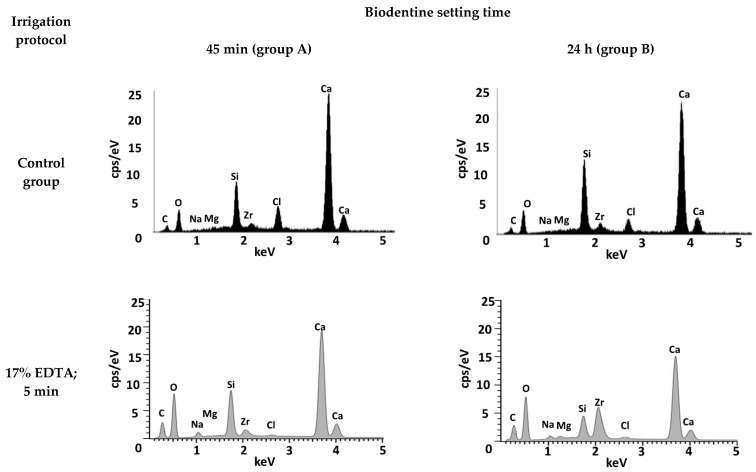

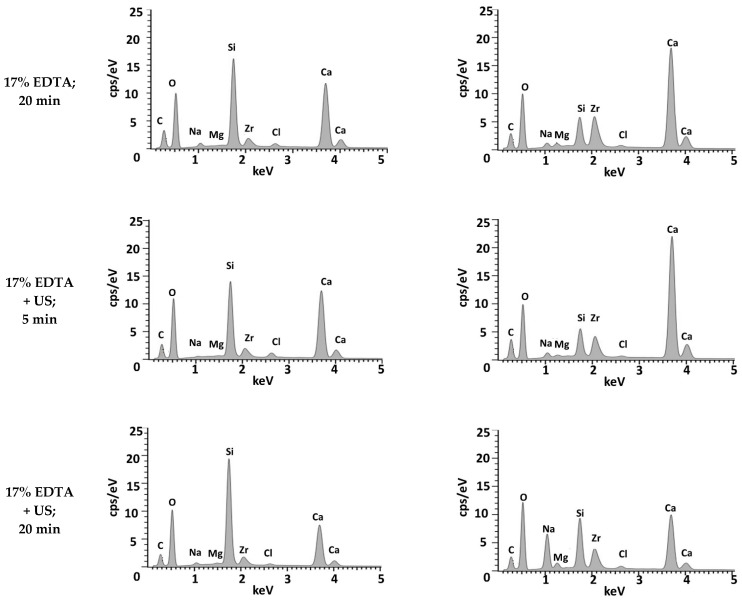
EDS spectrograms of Biodentine specimens.

The chemical percentage composition of each Biodentine specimen’s surface was registered on the basis of the data obtained with the EDS method (Table 1, Figure 3) and spectrograms were created (Figure 4). The results were compared between the groups and with the control group.

The EDS study results indicate the presence of elements specific to Biodentine—peaks of calcium, silicon, carbon, zirconium, chlorine and oxygen are clearly visible on the spectrograms.

The results show that the irrigation protocol with EDTA changes the chemical composition of the tested material and differences between the control and the investigated groups occur. Changes are also detectable when comparing the specimens after two setting times (group A and B) and the specimens subjected to ultrasonic irrigation.

Within specimens from group A, a trend was observed in the percentage of calcium. Its content decreased with the elongation of the rinsing time and after ultrasonic activation in favor of an increase in the percentage of silicon. The most pronounced change in the proportion of calcium was visible in the specimens from group B subjected to the longest rinsing protocol with US activation. In specimens from both setting time groups, an increasing percentage of zirconium was noted, with a clearer tendency in the specimens after the longer setting time (group B). It can therefore be concluded that both the longer time of irrigation and the use of ultrasonic activation enhance the chelating effect of ethylenediaminetetraacetic acid. The longer the setting time of Biodentine cement, the better its resistance to this action.

In comparison with the control group, the effect of irrigation protocols using 17% sodium edetate can be seen in almost every specimen. Only the specimens evaluated after the shortest setting and rinsing time without ultrasonic activation are similar in composition to the control. In the group A, the tendencies of changes are clear—the prolonged rinsing time with ethylenediaminetetraacetic acid as well as the use of ultrasonic activation decrease the percentage of calcium and chlorine, in opposition to zirconium and silicon in the surface composition of Biodentine. In specimens from group B, a decrease in the content of chlorine and silicon and an increase in zirconium percentage is detectable. A simultaneous decrease in the calcium level was only noticed in Biodentine specimens after the longest setting and rinsing time with ultrasonic activation.

### 3.2. Biodentine Surface Appearance

**Figure 5:**
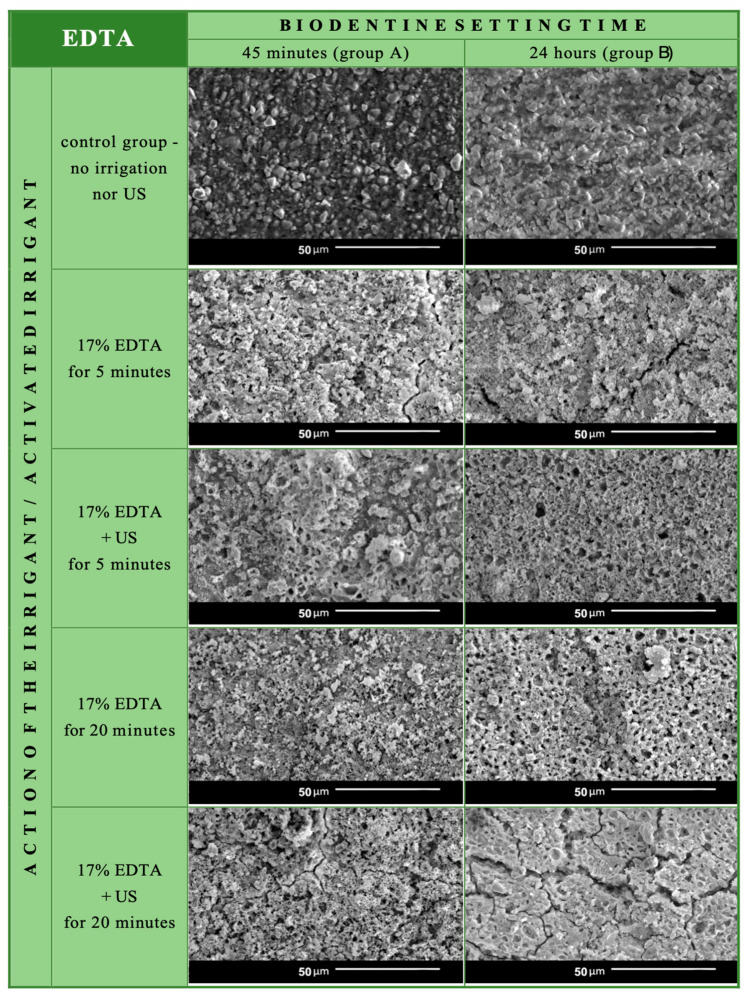
Biodentine specimens’ surface visualized in SEM.

SEM images of Biodentine specimens from both setting time groups were compared. The obtained results indicate the influence of 17% EDTA solution on the surface appearance of the investigated material when compared with the control specimens (Figure 5).

The effect of the irrigation protocol with 17% EDTA solution was visible on each tested specimen. The Biodentine surface was more irregular than in the control group. The material with hollow pits and round-shaped defects resembled the etched dentin with open tubules.

Specimens with longer irrigation exposure time were more affected than those after 5 min of rinsing. The ultrasonic activation also enhanced the chelating action of the tested irrigant. Although this result was noticeable in both setting time groups, Biodentine after 24 h setting seemed more susceptible to liquid action.

In both groups A and B, the longest irrigation time with ultrasonic activation resulted in the most pronounced changes on the surface of the cement. It became very irregular, with crystals “torn” from its surface.

### 3.3. Biodentine Surface Volume Loss

**Figure 6:**
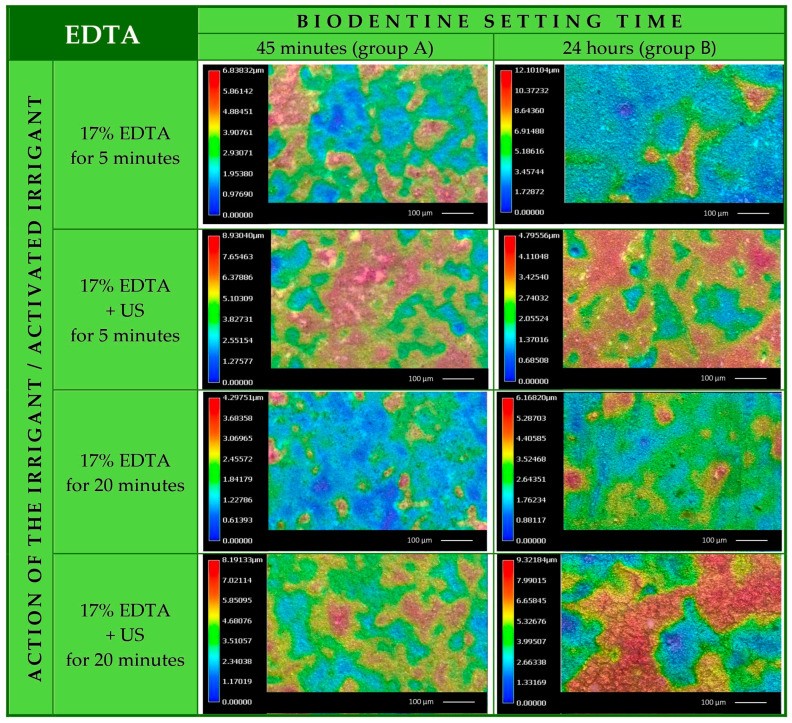
2D color map of Biodentine surface of the specimens from group A and B after irrigation with 17% EDTA with and without ultrasonic activation.

The specimens’ surface visualized as 2D color maps are shown in Figure 6.

The mean values of total volume loss, volume loss per 1 µm^2^ and standard deviation of each Biodentine specimen are presented in Table 2.

In the specimens from group A, the average volume loss after 5 min irrigation with EDTA was 3.98 µm^3^ for each µm^2^ of surface. After 5 min of rinsing with ethylenediaminetetraacetic acid with ultrasonic activation, the loss was higher: 7.74 µm^3^/µm^2^. For specimens subjected to a 20 min EDTA irrigation, the loss was 2.95 µm^3^ per µm^2^, whereas ultrasonic activation increased this value to 6.57 µm^3^/µm^2^.

In the specimens from group B, the loss of volume was increasing with the increase in rinsing intensity. After 5 min of EDTA irrigation, the volume loss was 6.30 μm^3^ for each μm^2^ of surface area. After 5 min with ultrasonic activation, the loss was higher: 11.70 µm^3^/µm^2^. For specimens subjected to 20 min irrigation with sodium edetate, the volume loss was 10.64 µm^3^ per µm^2^, and ultrasonic activation increased this value to 32.71 µm^3^/µm^2^.

Comparing the results to the baseline situation (control group without any irrigation), there was practically no effect of EDTA irrigation on the specimens from group A or on the specimen from group B rinsed for 5 min without US activation.

**Figure 7:**
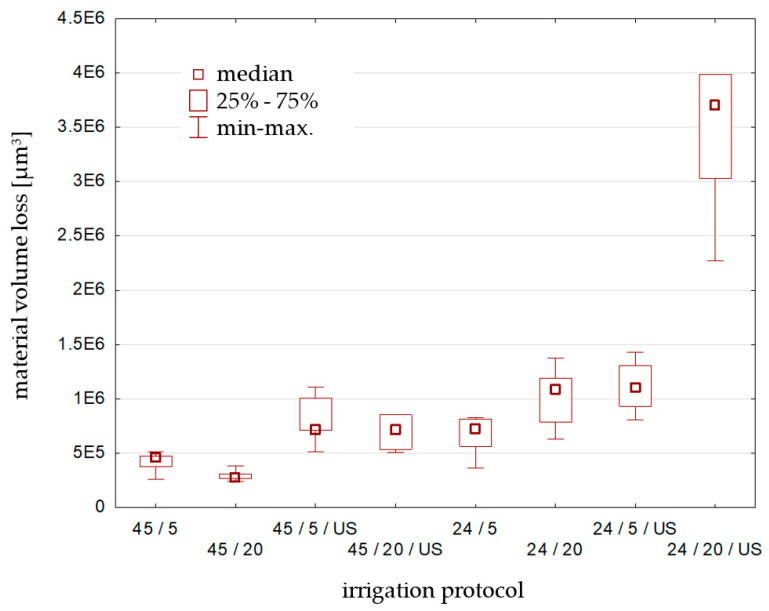
Statistical analysis—graphical comparison of the effect of different irrigation protocols using 17% EDTA on the volume loss of Biodentine. (45/5 = 45 min setting time, 5 min irrigation; 24/20/US = 24 h setting time, 20 min irrigation with ultrasonic activation etc.).

The statistical analysis of the obtained results is presented in Table 3 and in Figure 7 and shows the dependance of material volume loss from the chosen irrigation protocol. The Kruskal–Wallis test was used, where the volume loss [µm^3^] was the dependent variable and the irrigation protocol—the independent variable. Statistically significant differences were marked (*p* < 0.05000).

The results of the Kruskal–Wallis test (*p* = 0.0001), at the assumed level of significance (α = 0.05), indicate the rejection of the null hypothesis.

Statistically significant differences were found between borderline specimens: 45 min setting time, 5 min vs. 24 h setting time, 20 min irrigation with 17% EDTA ultrasonically activated as well as the Biodentine specimen from group A, after 20 min EDTA irrigation vs. specimens from group B after 20 min and 5 min EDTA irrigation with US activation. No clear trend was detected for any tested irrigation protocol.

## 4. Discussion

Biocompatibility and sealing properties of the modern biocements used in endodontics are the crucial features corresponding to the quality of the treatment and its success rate. Nevertheless, the material management is equally significant. It is essential for a clinician to know about the indications, adequate management and application techniques of biomaterials to ensure long-term treatment success. This research is therefore clinically relevant.

A perforation repair material should have adequately high dislodgement resistance to withstand the functional forces and forces of condensation [26]. The contact of perforation repair material with various irrigants and blood can inadvertently modify their physical properties and consequently affect the treatment outcome. According to Sethi et al., Biodentine showed maximum cohesive failures (62.5%) followed by mixed (20.8%) and adhesive failures (16.7%) [27]. The presented analysis of the cement features seems to confirm that observation. In the literature, the shear bond strength and push-out bond strength tests are the most often proposed to assess the dislodgement resistance of perforation repair materials in endodontics. The connection quality between Biodentine and dentin and its compressive strength or color stability are widely evaluated [28,29]. However, the impact of canal rinsing agents and their ultrasonic activation on Biodentine surface is less likely described. In some studies, the EDTA was not even included in the tested irrigation protocols [30]. Measurement of the volume loss of the material after the irrigation protocol with this chelator is the novelty of this trial.

Biodentine specimens were segregated on the basis of selected setting times [17]. The setting time recommended by the manufacturer—12 min [31]—was verified as too short for the analysis. The material was not set enough to withstand the protocol of irrigation and became completely disintegrated during the irrigation. That fact has made it impossible to continue the experiment in this group of specimens. Although it is supposed to be sufficient in conservative dentistry procedures [32,33], the degradation of the material surface may have a negative effect on the endodontic therapy outcome. After 12 min of setting, the cement was not yet able to withstand the immediate irrigation protocol, especially with US activation.

Rinsing with chelators is essential for the removal of the smear layer in the final part of chemo-mechanical cleaning and shaping of the canal space. From the clinical point of view, chelating irrigation liquids should be in contact with the dentin for no longer than 3 min in order to reduce canal wall erosion [34,35]. Nevertheless, in calcified, thin and curved canals, EDTA in the form of liquid, cream, gel, paste, etc. is used to obtain the patent canal and to enhance its mechanical cleaning and shaping [36]. Thus, the contact time of the chelator with the dentin and the bioceramic material placed in the endodontic space may be much longer. Therefore, the choice of prolonged irrigation times for this trial is still justified. Moreover, the presented results are part of the bigger project, comparing the data of the action of other agents, e.g., sodium hypochlorite. It was also the reason for choosing longer times of irrigation protocols (5 and 20 min). Group A consisted of Biodentine specimens set in a 45 min irrigation time, as indicated by Grech et al. [37]. After EDTA irrigation, the surface was visually affected. The material did not seem to be fully set, which can be explained by the standard behavior of cement and its setting mechanism. In accordance with the American Society for Testing and Materials (ASTM) standards, it is divided into two stages: initial setting and maturation or final setting. The initial phase lasts until the material starts losing its plasticity. The final phase ends when the cement becomes rigid, which means that with the application of increasing stress, it fractures rather than flows [38]. Biodentine derives from Portland cement, so its setting behavior resembles much the above described.

The material in group B was setting for a longer time, yet still the irrigation with 17% EDTA solution caused partial disintegration of the material surface. The ultrasonic activation as well as the increase in irrigation time seemed to amplify the EDTA effect on Biodentine more visibly than in specimens from group A. Taking the presented results and all of the above into consideration, it can therefore be concluded that both the longer time of irrigation and the use of ultrasonic activation enhance the chelating effect of ethylenediaminetetraacetic acid. The longer the setting time of Biodentine cement, the better its resistance to this action.

Numerous cracks could be noticed on the material surface visualized in SEM. Most probably, they were created as a result of the testing procedure, which involved obtaining a vacuum in the SEM chamber. This did not affect the volume microscopy measurements, as the tested spots on the Biodentine surface were consciously chosen by the operator.

The interpretation of EDS analysis needs to be carried out bearing in mind that the software equalizes the results up to 100%. This means that the increased percentage of a certain component leads to the decreased percentage of another, but not necessarily the actual decrease in number or volume. Taking into account the chelating nature of the tested irrigant, the loss of calcium is obvious. EDTA incorporates the mineral ion into a hydrosoluble complex or chelate [39]. The consequential rise in silicon level arises from the chemical breakdown of the tricalcium silicates formed by both elements. After irrigating the set material with EDTA, with the partial removal of calcium from the system comes the need to equalize the overall percentage to 100% by the software; hence, the increase in the calculated amount of silicon.

The obtained modifications in the percentage of elements in the surface composition of the tested specimens are only a preliminary indicator of the changes that occur in the material. The volume loss analysis included various measurement points on the specimens, which makes the statistics valid; nevertheless, obtaining data from a greater number of specimens is still a direction for further research.

This study is limited by its ex vivo form. An observation and analysis of the clinical behavior of Biodentine in endodontic procedures following perforation closure is highly advisable as a direction of future research.

## 5. Conclusions

Irrigation with EDTA solution, either ultrasonically activated or not, visibly affected the surface appearance of Biodentine. The chemical composition of the tested material was also altered. The effect is more visible after US activation of the irrigant. That is why the continuation of endodontic procedures involving irrigation with EDTA straight after Biodentine placement is not recommended. Further research is needed to evaluate the potential of the material changes described in this paper to alter the clinical outcome of the treatment.

## Figures and Tables

**Table 1:** Atom percentage [%] of Biodentine specimens’ surface in control group, group A (after 45 min setting time) and in group B (after 24 h setting time).

Element	45 min Setting Time					24 h Setting Time				
	No Irrigation	17% EDTA 5 min	17% EDTA 20 min	17% EDTA + US 5 min	17% EDTA + US 20 min	No Irrigation	17% EDTA 5 min	17% EDTA 20 min	17% EDTA + US 5 min	17% EDTA + US 20 min
Carbon	11.35	15.0	21.1	17.5	18.8	10.41	18.0	15.5	15.8	14.9
Oxygen	49.61	44.1	44.2	48.1	46.3	49.93	41.9	44.7	44.6	44.0
Silicon	5.96	7.0	12.5	11.1	17.2	9.09	3.1	3.6	3.4	7.5
Zirconium	0.56	2.5	3.2	3.4	3.8	0.74	1.,5	9.7	6.4	7.7
Chlorine	3.64	0.3	0.6	0.9	0.3	2.09	0.5	0.3	0.2	0.5
Calcium	28.87	29.9	17.5	18.7	12.9	27.74	23.4	24.2	28.0	15.2
Natrium	0.0	1.2	0.9	0.3	0.6	0.0	0.8	1.1	1.2	8.8
Iron	0.0	0.0	0.0	0.0	0.0	0.0	0.3	0.3	0.2	0.3
Aluminum	0.0	0.0	0.0	0.0	0.1	0.0	0.0	0.0	0.0	0.1
Magnesium	0.0	0.0	0.0	0.0	0.0	0.0	0.4	0.6	0.3	1.1

**Table 2:** The mean values of total volume loss, volume loss per 1 µm^2^ and standard deviation [µm^3^] in Biodentine specimens after EDTA irrigation with or without US activation.

Parameter	Irrigation Protocol				
	No Irrigation (Control)	17% EDTA 5 min	17% EDTA 20 min	17% EDTA + US 5 min	17% EDTA + US 20 min
Group A—45 min Setting					
total volume loss	602,927.89 µm^3^	417,313.69 µm^3^	295,658.41 µm^3^	811,488.96 µm^3^	692,396.96 µm^3^
volume loss per 1 µm^2^	5.03 µm^3^	3.98 µm^3^	2.95 µm^3^	7.74 µm^3^	6.57 µm^3^
standard deviation	298,351.33 µm^3^	99,037.94 µm^3^	54,593.00 µm^3^	242,789.08 µm^3^	166,958.76 µm^3^
Group B—24 h Setting					
total volume loss	602,927.89 µm^3^	660,091.36 µm^3^	1,012,729.76 µm^3^	1,115,836.11 µm^3^	3,394,783.66 µm^3^
volume loss per 1 µm^2^	5.03 µm^3^	6.30 µm^3^	10.64 µm^3^	11.70 µm^3^	32.71 µm^3^
standard deviation	298,351.33 µm^3^	196,107.81 µm^3^	302,416.00 µm^3^	256,712.78 µm^3^	738,214.44 µm^3^

**Table 3:** Statistical analysis—comparison of the effect of different irrigation protocols with 17% EDTA on the volume loss of Biodentine. Statistically significant differences (*p* < 0.0500) marked in italics.

		Group A				Group B			
		17% EDTA, 5 min	17% EDTA, 20 min	17% EDTA + US, 5 min	17% EDTA + US, 20 min	17% EDTA, 5 min	17% EDTA, 20 min	17% EDTA + US, 5 min	17% EDTA + US, 20 min
group A	17% EDTA, 5 min		1.0000	1.0000	1.0000	1.0000	0.3591	0.1064	*0.0014*
	17% EDTA, 20 min	1.0000		0.5023	1.0000	1.0000	0.0750	*0.0183*	*0.0001*
	17% EDTA + US, 5 min	1.0000	0.5023		1.0000	1.0000	1.0000	1.0000	0.7695
	17% EDTA + US, 20 min	1.0000	1.0000	1.0000		1.0000	1.0000	1.0000	0.2533
group B	17% EDTA, 5 min	1.0000	1.0000	1.0000	1.0000		1.0000	1.0000	0.1623
	17% EDTA, 20 min	0.3591	0.0750	1.0000	1.0000	1.0000		1.0000	1.0000
	17% EDTA + US, 5 min	0.1064	*0.0183*	1.0000	1.0000	1.0000	1.0000		1.0000
	17% EDTA + US, 20 min	*0.0014*	*0.0001*	0.7695	0.2533	0.1623	1.0000	1.0000	

## Data Availability

Data are contained within the article.
